# Effect of health insurance on delivery care utilization and perceived delays and barriers among southern Thai women

**DOI:** 10.1186/1471-2458-11-510

**Published:** 2011-06-28

**Authors:** Tippawan Liabsuetrakul, Nurlisa Oumudee

**Affiliations:** 1 Epidemiology Unit, Faculty of Medicine, Prince of Songkla University, Hat Yai, Songkhla 90112, Thailand

## Abstract

**Background:**

Financial reform aims to overcome the problems of financial barriers and utilization of health services. However, it is unclear whether financial reforms or health insurance can reduce delays and/or barriers or if there are still other important obstacles for preventing pregnant women accessing delivery care. This study aimed to assess the effect of health insurance and other factors on delivery care utilization and the perception of delays and barriers to delivery care among women living in Songkhla province, Thailand.

**Methods:**

A cross-sectional study was conducted from November 2007 to December 2008. Women who delivered at hospital or home in the areas of participating hospitals in four districts were interviewed at 24- or 48-hours postpartum. The impact of health insurance and other factors on outcomes of interest was assessed using multivariate logistic regression.

**Results:**

Of 2,847 women, 2,822 delivered at a hospital and 25 at home, of which 80% and 40% had health insurance for delivery care, respectively. Muslims, low educated women, those who thought they could not use health insurance for delivery care and those less willing to seek care at their delivery place were more likely to give birth at home. Perception of delays to seeking care, reaching a hospital and receiving care was reduced in women insured by civil servant medical benefit. Women insured by universal coverage and social security perceived a lower delay in reaching a hospital but a higher delay in receiving care. Low education, unwillingness to seek care, out-of-pocket payment, worry about cost of delivery care, transportation difficulties, low perception of receiving good care or a perception of being treated badly were also associated with delays and barriers to health care. Almost all (93%) agreed that health insurance could reduce financial barriers for accessing services. However, having health insurance influenced them to seek care, reach a hospital, and receive care quickly in 50%, 32%, and 23% of the women, respectively.

**Conclusions:**

Health insurance has a significant impact on perceived delays and barriers, but not place of delivery. Socio-economic determinants continue to play an important role for place of delivery and perceived delays and barriers.

## Background

According to WHO estimates, there were 358,000 maternal deaths worldwide in 2008 and 355,000 (99%) of these deaths occurred in developing countries [1]. The most common direct obstetric-related causes of death were hemorrhage, hypertension, unsafe abortion and sepsis [2]. Delivery by skilled birth attendants and the availability of facilities providing emergency obstetric care are cost-effective interventions for accomplishing the United Nations Millennium Development Goal (MDG) 5, which aims to improve maternal health by reducing the maternal mortality ratio and achieve universal access to reproductive health care by the year 2015 [1,3]. The three delays model, consisting of delay in seeking care, reaching a facility, and receiving adequate treatment at a facility, have been shown to be contributing factors to maternal mortality [4,5].

To access care at a health facility, pregnant women need to have sufficient finances to cover the costs of seeking care, transportation, and upfront fees for service, medication and other consumables [6]. Previous studies have demonstrated that financial constraints, geographic or distance challenges, transportation problems, lack of knowledge about the service facility, and/or the perception of a poor quality of care from the facility were the common barriers to delivery care utilization [7,8]. To ensure maternal health services and the achievement of the MDG 5, a country must have an effective system to support accessible and available delivery care services for pregnant women [6].

Health sector reform is defined as the actions taken by governments with a view to improving the performance of health care systems. The reform often primarily emphasizes the financing and organization of the service [9]. Due to the world economic crisis in 1997, the health system in Thailand also faced financial crisis, demographic and epidemiological changes, and an increasing demand for services. In 2000, the coverage of health insurance in Thailand consisted of the Civil Servant Medical Benefit Scheme (CSMBS) for government employees and state enterprise employees, the Social Security Scheme (SSS) and Workman's Compensation Fund for private sector employees, Health Card Fund and Fee Exemption for the poor and underprivileged, and private insurance. About 20% of the population was uninsured. To overcome the problems facing the uninsured and the financial barriers for the poor and underprivileged, the Universal Coverage Scheme (UCS) was developed in 2001 and health insurance in Thailand was reformed and currently there are three main health insurance schemes, namely CSMBS, SSS and UCS [10]. All insurance schemes cover the costs of medical care for delivery services in public hospitals; however, SSS and UCS cover services in registered hospitals and the costs of the first two births only [11].

Despite this reform to overcome financial barriers and increase utilization of health care services, to date there has been no quantifiable evaluation in terms of consumers' perspectives, especially concerning delivery care [12]. The objectives of this study were to determine whether health insurance reforms have had an influence on utilization of delivery care and perception of delays and barriers in accessing delivery care.

## Methods

### Study settings and samples

A cross-sectional study was conducted in Songkhla province of southern Thailand from November 2007 to December 2008. Songkhla is one of the largest provinces in the southern region and there is a mixture of ethnic, cultural, and religious groups. Although Songkhla province has developed into one of southern Thailand's major urbanized provinces, where residents have a certain freedom to receive services at their preferred hospital, the rate of home-based delivery has been reported to be as high as 10% [13]. As a result of this rate of home delivery and amalgam of its population, Songkhla was selected to be the study setting.

All women who delivered at the participating hospitals or at home in the vicinity of the participating hospitals were included in this study. Non-Thai citizens, those who could not be interviewed within 48 hours postpartum due to severe complicated conditions, and those who died during labor or delivery were excluded from the study. Four of eight district hospitals where the number of deliveries was approximately 30-50 women per month, were randomly selected using computer-generated random numbers. One regional hospital and one university hospital in the study setting in were also selected.

Due to the unavailability of information related to perception of delays or barriers in accessing hospital-based delivery care, the sample size of women who delivered in district hospitals was calculated according to an estimated proportion of any delays or barriers to service utilization of 15%. In addition, a proportion of obstetric complications of 25% in referral hospitals were considered. Assuming 10% rate of incomplete data, a total of 220 women in each district hospital and 875 women in each referral hospital were needed to estimate the above proportions to a precision of 5%.

### Preparatory phase

Before the study was conducted, the proposal was presented to the chief medical officers of the Songkhla Provincial Health Offices and the hospital directors of the study hospitals for their approval. Research assistants who were not the personnel of the hospital were trained to be the interviewer. To achieve similar standards in interviewing techniques and data collection processes two full-day workshops were conducted separately: one for training the interviewers (research assistants and local health personnel) and the other for nurses who helped to facilitate interviews at the hospitals. The contents of each workshop included the introduction of the proposal, objectives of the interview, details of the questionnaire, and techniques for the interview and all data collection processes.

### Data collection phase

All women who gave birth at study hospitals and were admitted to the postpartum ward were approached by a trained interviewer at 24- or 48-hours postpartum. After agreeing to participate in the study, they were interviewed in a private area where their responses were not heard by hospital staff. In addition, their obstetric data were collected from the medical records.

In southern Thailand, all pregnant women in the local community are monitored by the local health personnel and all births at home are recorded. In general, all women who give birth at home are assisted by a traditional birth attendant (TBA). In this study, all women who gave birth at home were visited by local health personnel who worked at the local health center and who had no close relationship with the TBA. Those who agreed to join the study were interviewed at a time of their convenience and separate from their family members. They were also informed that the TBA neither saw nor heard their responses.

After the interviews, the signed consent forms and questionnaires were placed in a sealed envelope, signed across the flap by the interviewee, and placed in a locked filing cabinet at the hospital or health center. For women who gave birth at home, data on obstetric conditions and complications in pregnancy and delivery were obtained during the interview.

### Variables

The dependent variables were the utilization of hospital-delivery care by pregnant women and perceived delays and barriers in (i) their decision to seek care, (ii) reaching a hospital, and (iii) receiving care at hospital. Although this "three delays" model has been widely cited for evaluating maternal death [4,5], the definitive classifications for delays are not specified objectively. In our study, women's perception on delay was categorized as *very late, late, intermediate, early*, and *very early*, and then dichotomized as either delay (*very late, late, intermediate*) or no delay (*early, very early*). Perceived barrier was measured by directly asking the women whether any factors related to finance, local customs, religion, beliefs, geography, transportation, type of hospital, and civil unrest [7,8] prevented them from making a decision to seek care, reach a hospital, and receive care at a hospital. Each barrier was classified as a perceived barrier, if their response was "yes" or an unperceived barrier if their response was "no".

Independent variables were age, occupation, religion, education, monthly family income, parity, type of payment for their delivery care (Universal Coverage Scheme (UCS), Social Security Scheme (SSS), and Civil Servant Medical Benefit Scheme (CSMBS) or Out-of-Pocket), women's perception on the coverage of their health insurance for delivery care and other potential factors.

Potential individual factors associated with perceived delays and barriers to seeking care included chief medical complaints, willingness to seek care at their delivery place (on a rating scale of 1 to 5, where 1 means not willing at all and 5 means absolutely willing), family support for delivering in hospital, concern about civil unrest, transportation difficulty if symptoms occurred at night, worry about the cost of delivery care. Hospital factors included type of hospital, proximity of hospital to home, hospital regulation supporting delivery care, companionship during labor, companionship during delivery and permission to perform religious rituals.

Potential factors for perceived delays and barriers to reaching a hospital were conditions of the road, means of transportation to hospital, transportation management problems and transportation difficulty due to civil unrest. Potential factors for perceived delays and barriers to receiving care at hospital were the perception of receiving good care and thoughts of being treated badly. The effect of health insurance on helping to reduce the barriers and influence their early decision to seek, reach, and receive care was asked directly to each woman.

### Data management and analysis

Data obtained from the questionnaires were coded and entered into EpiData software version 3.1. R software was used for data analysis (the R Foundation for Statistical Computing 2009, Austria).

The characteristics of women who gave birth were described as percentages and factors associated with place of delivery were assessed by fitting a multiple logistic regression model with the positive outcome being delivery at home. Direct responses of women on the effect of health insurance to reduction of barriers and their decision to seek care, reaching a hospital, and receiving care were analyzed descriptively. The effects of health insurance on the perceived delays and barriers for seeking, reaching and receiving care were also assessed using a multiple logistic regression model with stepwise backward methods. The significance of variables was determined using Wald's test where a p-value less than 0.05 was considered as statistically significant.

### Ethical considerations

Ethical approval for the study was obtained from the Institute Ethical Review Committee of the Faculty of Medicine of Prince of Songkla University, Hat Yai, Songkhla, Thailand (SUB.EC 50/370-026 on August 1, 2007) and the Ministry of Public Health (Document No. 100/2007 on September 26, 2007).

## Results

A total of 3,229 women delivered during the study period, of which 3,204 women gave birth in hospital and 25 gave birth at home.

All 25 women who gave birth at home were interviewed. Of the 3,204 women who delivered in hospital, 382 (12%) were not recruited due to time constraints (5.6%), technical difficulties (4.2%), non-Thai citizenship (1.6%), complicated conditions (0.2%), and refusal to participate (0.4%). Time constraints occurred in the two referral hospitals where the number of deliveries was high and the time required for interview was limited.

### Delivery care utilization: home-based versus hospital-based delivery

Table 1 shows the factors associated with hospital- or home-based delivery by univariate analysis. Occupation, religion, education, monthly family income, parity, type of payment, women's perception on the coverage of their health insurance for delivery care, and their willingness to seek delivery care at their delivery place were significantly different. Women who gave birth at home had a significantly higher proportion of unemployed, Muslims, less educated, lower income earners, women who gave birth more often, women who were more likely to pay for delivery out-of-pocket, those who perceived their health insurance to not cover the cost of delivery care and those less willing to seek care at their delivery place.

**Table 1:** Comparison of factors for hospital- and home-based deliveries by univariate analysis

Characteristics	Hospital N = 2,822	Home N = 25	p-value*
**Age group **(n = 2846)			0.40
<20	328 (11.6)	5 (20.0)	
20-24	747 (26.5)	4 (16.0)	
25-34	1317 (46.7)	11 (44.0)	
≥35	429 (15.2)	5 (20.0)	
**Occupation **(n = 2847)			<0.001
Housewife or Student	831 (29.4)	11 (44.0)	
Government employee	294 (10.4)	0	
Company employee	356 (12.6)	0	
Gardener	547 (19.4)	13 (52.0)	
Laborer	439 (15.6)	0	
Merchant	355 (12.6)	1 (4.0)	
**Religion **(n = 2847)			<0.001
Buddhism	1933 (68.5)	2 (8.0)	
Islam	875 (31.0)	23 (92.0)	
Christianity	14 (0.5)	0	
**Education **(n = 2846)			<0.001
Illiterate	77 (2.7)	0	
Primary school	632 (22.4)	22 (88.0)	
Secondary school	1430 (50.7)	3 (12.0)	
Bachelor's degree or higher	682 (24.2)	0	
**Monthly family income (USD) **(n = 2838)			0.004
No income	22 (0.8)	0	
330 or less	1110 (39.4)	18 (72.0)	
More than 330	1681 (59.8)	7 (28.0)	
**Parity **(n = 2847)			<0.001
0	1314 (46.6)	4 (16.0)	
1-2	1310 (46.4)	10 (40.0)	
3-4	162 (5.7)	8 (32.0)	
5 or more	36 (1.3)	3(12.0)	
**Type of payment **(n = 2847)			<0.001
UCS	1186 (42.0)	11 (44.0)	
SSS	737 (26.1)	0	
CSMBS	354 (12.6)	0	
Out of pocket	545 (19.3)	14 (56.0)	
**Women's perception on coverage of health insurance for delivery care **(n = 2831)			<0.001
No	428 (15.3)	15 (60.0)	
Yes	2378 (84.7)	10 (40.0)	
**Willingness to seek care at their delivery place **(n = 2844)			<0.001
Low	88 (3.1)	12 (48.0)	
High	2731 (96.9)	13 (52.0)	

UCS: Universal Coverage Scheme; SSS: Social Security Scheme; CSMBS: Civil Servant Medical Benefit Scheme; *Fisher's exact test

No woman who gave birth at home was insured by SSS or CSMBS so a new dichotomous variable was created and designated as insured or not insured (paid out-of-pocket) for inclusion in the logistic regression model. Results of the final model for associated factors between hospital and home deliveries are shown in Table 2. Muslims, women with a primary school education or less, those who perceived that their health insurance would not cover the cost of their delivery care and those who had a low willingness for seeking care at their delivery place were significantly more likely to give birth at home. After adjusting for these factors in the final logistic regression model, health insurance had no significant effect on place of delivery (adjusted OR 0.7; 95% CI 0.1-4.7).

**Table 2:** Final logistic regression model for associated factors of home-based delivery compared to hospital-based delivery

	Final model		
	
Variables	Crude OR (95% CI)	Adjusted OR (95% CI)	p-value*
**Type of payment **(Ref = Insured^**†**^)			
Out of pocket	6.4 (2.9-14.4)	0.7 (0.1-4.7)	0.69
**Religion **(Ref = Buddhism)			
Islam	25.7 (6.0-109.3)	35.0 (7.0-174.2)	<0.001
**Education **(Ref = Secondary school or higher)			
Primary school or less	22.2 (6.6-74.3)	29.0 (7.0-120.0)	<0.001
**Perception on the use of health insurance for delivery care **(Ref = Yes)			
No	8.4 (3.7-18.7)	26.1 (3.5-193.8)	0.001
**Willingness to seek care at delivery place **(Ref = High)			
Low	30.1 (13.4-68.0)	37.7 (12.3-115.9)	<0.001

*Wald's test

^**†**^Only universal coverage was available for health insurance of women who delivered at home

OR: Odds ratio; CI: Confidence Interval

### Home-based delivery

Of the 25 women who delivered at home, reasons for their decision to deliver at home included thoughts of inability to reach a hospital in time (n = 18), transportation problems due to symptoms beginning at night (n = 15), proximity to the hospital (n = 12), family preference (n = 10), thought of unaffordable costs (n = 4), and concern about the civil unrest in the area (n = 3). A woman could give more than one reason. Twenty said that their decision to deliver at home was their own and 5 were influenced by other family members. The rate of prenatal care among these women was 80%. Twenty four delivered at home with the assistance of a TBA and one woman was assisted by a village health volunteer. All mothers and babies were free of complications, except for one baby which was born with club feet and was referred to hospital.

### Delays and barriers on seeking, reaching and receiving care: Hospital-based delivery

#### Seeking care

The perception of delays and barriers to seeking hospital-based delivery care is shown in Table 3. Women insured by CSMBS were significantly less likely to perceive a delay and barrier to seeking delivery care. Women who had a family income of 330 USD or less and those who were unwilling to seek care at their delivery place were more likely to perceive delays and barriers to seeking care. Women who thought that they were unable to afford delivery care were more likely to perceive that barriers existed preventing them from seeking delivery care. In contrast, they were less likely to perceive delays in seeking delivery care. Ongoing civil unrest in the study area as well as transportation difficulties caused an increased likelihood of perceived delay while women who believed that hospitals whose regulations supported delivery care and allowed them to practice religious rituals were less likely to perceive delays in seeking delivery care.

**Table 3:** Final logistic regression model identifying factors associated with the perception of delays and barriers to seeking care for hospital-based delivery

Factors	Delay in seeking care N = 2390		Barrier to seeking care N = 2818	
	
	Adjusted OR (95% CI)	p-value*	Adjusted OR (95% CI)	p-value*
**Type of payment **(Ref = Out-of-pocket)				
UCS	1.2 (0.9-1.6)	0.15	0.9 (0.7-1.2)	0.50
SSS	1.2 (0.9-1.5)	0.29	0.9 (0.7-1.2)	0.39
CSMBS	0.4 (0.3-0.7)	<0.001	0.6 (0.4-0.9)	0.006
**Religion **(Ref = Buddhism)				
Islam	1.24 (1.01-1.5)	0.04		
**Education **(Ref = Secondary school or higher)				
Primary school or less			1.8 (1.5-2.2)	<0.001
**Monthly family income **(Ref = More than 330 USD)				
330 or less	1.4 (1.1-1.6)	0.003	1.6 (1.4-2.0)	<0.001
**Parity **(Ref = Nulliparous)				
Multiparous	0.8 (0.6-0.9)	0.007		
Grand multiparous	1.2 (0.5-2.9)	0.63		
**Willingness to seek care at delivery place **(Ref = Yes)				
No	1.7 (1.04-2.7)	0.03	2.1 (1.3-3.3)	0.003
**Chief complaints **(Ref = None)				
Labor pain	0.4 (0.2-1.0)	0.06		
Rupture of membranes	0.4 (0.2-0.9)	0.02		
Mucous bloody show	0.2 (0.1-0.6)	0.002		
Indicated conditions*	0.5 (0.2-1.2)	0.12		
**Family support for hospital-base delivery **(Ref = Yes)				
No			2.6 (1.4-4.8)	0.002
**Concern about civil unrest **(Ref = No)				
Yes	2.0 (1.2-3.3)	0.01		
**Transportation difficulty if symptoms occurred at night **(Ref = No)				
Yes	1.9 (1.4-2.6)	<0.001		
**Worry about the cost of delivery care**				
Yes	0.5 (0.3-0.6)	<0.001	2.6 (2.0-3.3)	<0.001
**Consider type of hospital for delivery **(Ref = No)				
Yes			1.4 (1.2-1.8)	<0.001
**Proximity of hospital **(Ref = Far from home)				
Near to home	0.8 (0.6-1.0)	0.02	0.6 (0.5-0.7)	<0.001
**Hospital regulations supporting delivery care **(Ref = no)				
Yes	0.5 (0.4-0.7)	<0.001	0.8 (0.6-1.0)	0.03
**Permission to perform religious rituals **(Ref = No)				
Yes	0.6 (0.4-0.8)	<0.001		

^**†**^Not asking for perception of delay to seek care due to no time of decision with appointment for admission or admission at/after ANC (n = 429; missing data, n = 3)

*Indicated conditions: fresh bleeding per vagina/decreased fetal movement/postterm

UCS: Universal Coverage Scheme; SSS: Social Security Scheme; CSMBS: Civil Servant Medical Benefit Scheme; OR: Odds ratio; CI: Confidence Interval

#### Reaching care

Table 4 shows the results of fitting a logistic regression model to perception of delays and barriers to reaching a hospital for delivery care. The likelihood of perceived delays was significantly less for women who were insured by UCS and SSS, higher for women whose monthly family income was 330 USD or less and women who had a vehicle but experienced transportation management problems. No effect of any health insurance compared to out-of-pocket payment was found in the perceived barrier to reaching care. Low education, road inconvenience, and not having a vehicle of their own increased the likelihood of perceiving barriers to reaching care.

**Table 4:** Final logistic regression model identifying factors associated with the perception of delays and barriers for reaching hospital care

Factors	Delay in reaching care N = 2815		Barrier to reaching care N = 2818	
	
	Adjusted OR (95% CI)	p-value*	Adjusted OR (95% CI)	p-value*
**Type of payment **(Ref = Out-of-pocket)				
UCS	0.6 (0.4-0.8)	0.002	0.8 (0.6-1.1)	0.15
SSS	0.6 (0.4-0.9)	0.02	0.9 (0.6-1.2)	0.42
CSMBS	0.6 (0.4-1.1)	0.13	0.7 (0.4-1.1)	0.09
**Education **(Ref = Secondary school or higher)				
Primary school or less			1.7 (1.3-2.2)	<0.001
**Monthly family income **(Ref = More than 330 USD)				
330 or less	1.6 (1.1-2.2)	0.01		
**Condition of the road **(Ref = Good)				
Poor			3.6 (2.7-4.9)	<0.001
**Means of transportation to hospital **(Ref = Family/self-owned vehicle)				
Friend's vehicle			1.6 (1.2-2.0)	0.001
Public transportation			2.5 (1.6-3.9)	<0.001
**Transportation management problems **(Ref = No)				
No	2.8 (1.6-4.8)	<0.001		

UCS: Universal Coverage Scheme; SSS: Social Security Scheme; CSMBS: Civil Servant Medical Benefit Scheme; OR: Odds ratio; CI: Confidence Interval

*Wald's test

#### Receiving care

Table 5 shows the results of fitting a logistic regression model to perception of delays and barriers to receiving care at a hospital. Perceived delays of receiving care were significantly more likely to be detected in women who were insured with UCS and SSS but less likely to be in those who were insured with CSMBS. No effect of health insurance compared to out-of-pocket payment was found in barriers to receiving care. Women with low education had a higher likelihood of perceived barriers but a lower likelihood of perceived delays. Low family income, low perception of receiving good care or thoughts of being treated badly increased the likelihood of perceiving both delays and barriers to receiving care.

**Table 5:** Final logistic regression model identifying factors associated with the perception of delays and barriers for receiving care in hospital

Factors	Delay in receiving care N = 2815		Barrier to receiving care N = 2818	
	
	Adjusted OR (95% CI)	p-value*	Adjusted OR (95% CI)	p-value*
**Type of payment **(Ref = Out-of-pocket)				
UCS	1.4 (1.1-1.9)	0.009	1.3 (0.9-1.8)	0.14
SSS	1.4 (1.03-1.9)	0.03	1.0 (0.7-1.6)	0.80
CSMBS	0.5 (0.3-0.7)	<0.001	0.6 (0.4-1.1)	0.111
**Education **(Ref = Secondary school or higher)				
Primary school or less	0.8 (0.6-0.96)	0.02	2.0 (1.5-2.7)	<0.001
**Monthly family income **(Ref = More than 330 USD)				
330 or less	1.5 (1.2-1.9)	<0.001		
**Perception of receiving good care **(Ref = Yes)				
No	9.8 (8.1-12.0)	<0.001	1.6 (1.3-2.1)	<0.001
**Thoughts of being treated badly **(Ref = No)				
Yes	1.2 (1.0-1.5)	0.06	5.1 (3.9-6.6)	<0.001

UCS: Universal Coverage Scheme; SSS: Social Security Scheme; CSMBS: Civil Servant Medical Benefit Scheme; OR: Odds ratio; CI: Confidence Interval

*Wald's test

#### Effect of health insurance on delays

Almost all women (93%) said that health insurance could reduce their financial barrier, although their perception of health insurance to hasten their decision to seek care, reach a hospital, and receive care was found in only 50%, 32%, and 23% of women, respectively.

## Discussion

Utilization of hospital-based delivery care was high in Songkhla province, southern Thailand. Very few women delivered at home and nearly half perceived that the universal coverage health insurance scheme could pay for the cost of their delivery care. Nearly all of the women who gave birth at home were Muslims and low educated. The effect of health insurance was not significant in determining place of delivery, but it was significant in relation to the perceptions of delays and barriers in women who had hospital-based delivery, especially in those who were insured with CSMBS. Perceived delays or barriers were also affected by education and family income, willingness to seek care at their delivery place, concerns on civil unrest, family support for hospital delivery, geographic or transportation difficulties, and a perception of low quality of care.

Although high utilization of delivery care was noted in our study, there was inequality of access to health care since women who gave birth at home were mostly Muslim, low educated and had a lower family income than those who gave birth at a hospital. This finding supports the results of the Multiple Indicator Cluster Survey carried out by the National Statistical Office of Thailand from December 2005 to February 2006 which showed that the inequity of delivery by skilled birth attendants was related to economic, educational and geographic disparity [14]. However, the National Reproductive Health Survey, which was conducted in 2006 and 2009 in Thailand, reported a narrow gap of delivery by skilled birth attendants across geographic areas, women's level of education and household wealth [15].

Inequalities across coverage of hospital-based delivery are an international concern. Among Association of Southeast Asian Nations (ASEAN) countries, inequality was recently found to be substantial in the Philippines, Laos, and Cambodia, but not in Thailand or Vietnam [2]. A study in Vietnam found an overall rate of hospital-based delivery of 76.7% and the rate depended on number of prenatal visits, education level, income, number of children, and region of residence [16]. Similar factors were also identified in a study in Mali where the rate of hospital-based delivery was low (26.3%) [7]. This is supported by the social-system approach indicating that health services utilization depends on predisposing, enabling, and need factors [17].

Some women who delivered at home said they had intended to deliver at a hospital, but they were worried about not reaching the hospital in time due to a feeling of easy or imminent birth, a finding similar to a community-based study in Bolivia [8]. This worry can be minimized if the women received prior counseling on birth preparedness during prenatal care. The majority of participants in our study attended prenatal care, thus its effect could not be determined. In a study conducted in Maharashtra, India, parity and maternal age were found to be significant determinants for place of delivery, a finding not supported by our study [18]. In our study, place of delivery was mostly decided by the women themselves and this figure is supported by a previous study which found that the opinions of the husband did not contribute to the woman's decision to deliver at home [8].

Evidence from one study showed that the economic status and financial constraints of women were extremely important factors in their decision to deliver at hospital and in the utilization of skilled birth attendants in developing countries [19]. Of 75 countries for which Demographic and Health Surveys are available, the data showed that the increase of government sharing on health care spending was associated with the improvement of utilization and quality of delivery care, but not with prenatal care [20]. The economic constraints of women were identified to be one of the barriers to hospital-based delivery in Asia and Africa [21,22].

Women insured with CSMBS perceived lower delays in their decision to seek care, reach a hospital and receive care at a hospital than those who paid out-of-pocket. There are three possible explanations for this. Firstly, the benefit package of CSMBS is the most generous among the three types of health insurance schemes [11]. Secondly, the target population of this insurance consists of government employees and their wives who may be more educated and wealthier than women who are covered by SSS or UCS. Thirdly, those insured by SSS and UCS can receive the health services only in a registered hospital [10,11] and they might doubt whether they would receive care as adequate as the others leading to an increase of their perception of delay in receiving care in a hospital. Transportation convenience, friendliness of hospital staff, regulations for delivery care, and quality of care were also shown to be important determinants of perceived delays and barriers for accessing delivery care. Thus, these need to be improved in order to achieve universal access to health care.

The findings of this study contributed to the advancement of knowledge on the factors associated with delivery care utilization and the delays and barriers to utilization, in particular, the effect of financial reform on health insurance in women having hospital-based deliveries. There were some limitations in this study. Firstly, the number of women who delivered at home was small and less than expected, although we specifically selected the study settings where home births were monitored and extended the duration of data collection. Secondly, the outcomes of delays and barriers for seeking, reaching, and receiving care depended on women's own perceptions. However, as mentioned before, there is no standard definition to determine actual delay for accessing delivery care.

## Conclusions

Health insurance has a significant impact on perceived delays and barriers, but not place of delivery. Socio-economic determinants such as religion, education, monthly family income, willingness to seek care, geographic or transportation difficulties, concerns over civil unrest, financial constraints and quality of care are also crucial factors in the reduction of perceived delays and barriers to utilization of delivery care. The advantages of health insurance for universal access to delivery care services needs to be promoted to achieve the appropriate use of health insurance and aims of financial reform.

## Competing interests

The authors declare that they have no competing interests.

## Authors' contributions

TL designed the study, coordinated the data collection, analyzed and interpreted the data, and prepared the manuscript. NO coordinated the data collection and analyzed and interpreted the data. Both authors read and approved the final manuscript.

## Pre-publication history

The pre-publication history for this paper can be accessed here:

http://www.biomedcentral.com/1471-2458/11/510/prepub
